# Effects of a Multimodal Approach Using Buprenorphine with/without Meloxicam on Food Intake, Body Weight, Nest Consolidating Behavior, Burrowing Behavior, and Gastrointestinal Tissues in Postoperative Male Mice

**DOI:** 10.3390/vetsci9110589

**Published:** 2022-10-26

**Authors:** Kayo Furumoto, Yuka Sasaki, Masakatsu Nohara, Nagisa Takenaka, Noritaka Maeta, Teppei Kanda

**Affiliations:** Faculty of Veterinary Medicine, Okayama University of Science, Ikoino-oka 1-3, Imabari 7948555, Ehime, Japan

**Keywords:** multimodal analgesia, buprenorphine, meloxicam, analgesic effect, well-being, mouse

## Abstract

**Simple Summary:**

Distress affects animal welfare and the reliability of scientific data. Although preemptive analgesia and multimodal analgesia help minimize postoperative animal stress, optimizing well-being, there is a lack of reports on the effects and influence of multimodal approaches in mice. In this study, under the hypothesis that a multimodal analgesic protocol using buprenorphine with meloxicam has analgesic effects, we evaluated the effects of a multimodal analgesic protocol using buprenorphine with meloxicam on the well-being of mice during analgesic administration by changing the dosage of meloxicam. Mice were divided into nonoperative and operative groups and treated with an anesthetic (isoflurane) and analgesics (buprenorphine 0.1 mg/kg + meloxicam 0, 2.5, or 5 mg/kg). The evaluation parameters were body weight, food intake, animal behavior, and gastrointestinal tissue histology. When buprenorphine was combined with meloxicam, administering up to 5 mg/kg/day of meloxicam for 48 h to male mice (7 mice per group) after abdominal surgery had no significant negative effects on food intake; body weight; nest consolidating and burrowing behaviors; or gastric, duodenal, and jejunum tissue composition. In conclusion, a multimodal analgesic protocol of buprenorphine with meloxicam is among the options for increasing well-being in mice following abdominal surgery.

**Abstract:**

Distress affects animal welfare and scientific data validity. There is a lack of reports on the effects of multimodal analgesic approaches in mice. In this study, under the hypothesis that a multimodal analgesic protocol using buprenorphine with meloxicam has analgesic effects, we evaluated the effects of a multimodal analgesic protocol using buprenorphine with meloxicam on the well-being of mice during analgesic administration by changing the dosage of meloxicam. A total of 42 Slc:ICR male mice were categorized into nonsurgical and surgical groups (7 mice per group) and treated with an anesthetic (isoflurane) and analgesics (buprenorphine ± meloxicam). Analgesics were administered for 48 h after treatment. Buprenorphine (subcutaneous; 0.1 mg/kg/8 h) and meloxicam (subcutaneous; 0, 2.5, or 5 mg/kg/24 h) were administered twice. Body weight, food intake, nest consolidation score, and latency to burrow were evaluated. A significant decrease in food intake was observed 24 h after treatment, while a significant increase was observed 48 h post-treatment in all groups. Body weight showed a decreasing trend but was not significantly reduced. Furthermore, stomach, duodenum, and jejunum tissues showed no morphological abnormalities. Significant differences in burrow diving scores and the latency to burrow were observed between some groups, but these were not regarded as a consequence of the surgery and/or the meloxicam dose. When buprenorphine and meloxicam were combined, administering up to 5 mg/kg/day of meloxicam for 48 h to male mice after abdominal surgery had no significant negative effects on any tested parameters. In conclusion, a multimodal analgesic protocol of buprenorphine with meloxicam is among the options for increasing well-being in mice following abdominal surgery.

## 1. Introduction

Distress affects not only animal welfare but also the scientific validity of data obtained through animal experiments [1]. For ethical, social, and scientific reasons, researchers need to eliminate or minimize distress when conducting animal experiments [2,3]. Surgical procedures involving tissue damage can cause pain to animals. Preemptive analgesia and multimodal analgesia reduce postoperative stress in animals, optimizing the well-being of model animals [4,5,6]. Multimodal approaches combining opioids and nonsteroidal anti-inflammatory drugs (NSAIDs), including buprenorphine and indomethacin [7], buprenorphine and carprofen [8,9], fentanyl and paracetamol [10], as well as morphine and various NSAIDs have been reported in mice [11]. Here, we report a multimodal approach using buprenorphine and meloxicam in mice. Buprenorphine is a long-acting opioid [7,12], and meloxicam is a long-acting NSAID [12]. These two drugs were selected in a previous study with the aim of reducing distress in animals by decreasing the frequency of analgesic administration [13]. In our earlier report, we suggested that this multimodal analgesic protocol potentially relieves pain associated with open abdominal surgery [13]. Furthermore, we highlight that focusing merely on relieving surgical pain is inadequate as other types of distress must be considered to ensure animal well-being. The group of mice in the previous study that was anesthetized and treated with analgesics without surgery had the same negative side effects as the group that was anesthetized and treated with analgesics following surgery [13]. Since perioperative distress in laboratory animals is not limited to surgery-associated pain and since experimental procedures are also a potential cause of negative effects, the selection, dosage, and frequency of analgesics needs to be refined to minimize the effects of the various factors interfering with postoperative recovery in mice [13].

Under the hypothesis that a multimodal analgesic protocol using buprenorphine with meloxicam has analgesic effects, the purpose of our study was to evaluate the effects of a multimodal analgesic protocol using buprenorphine with meloxicam on the well-being of mice during analgesic administration by changing the dosage of meloxicam. The evaluation parameters were body weight, food intake, animal behavior, and gastrointestinal tissue histology. As in our previous studies, burrowing and nest-building behaviors were used to evaluate mouse behavior. These are innate behaviors and have been used to evaluate the well-being of mice [14,15,16]. Furthermore, these behavioral evaluations have been considered useful for assessing pain in postoperative mice [1,8,17,18,19,20,21,22].

## 2. Materials and Methods

### 2.1. Animals and Experimental Setup

**Figure 1:**
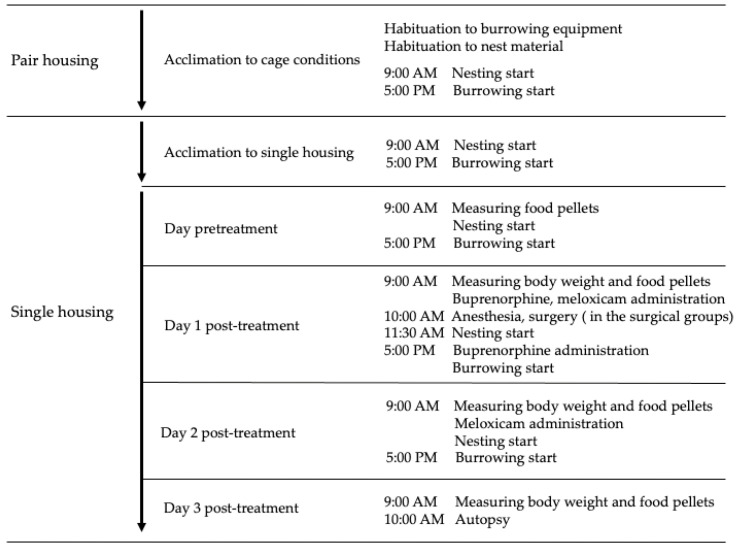
Outline of the experimental schedule.

Slc:ICR male mice (*n* = 42; 5-week-old) used in this study were obtained from SLC (Hamamatsu, Japan). The animal room was controlled at a temperature of 24–26 °C and relative humidity of 40–60%, under a 12 h light (7:00–19:00 h) and 12 h dark (19:00–7:00 h) cycle using artificial light. Mice were kept in polycarbonate cages (Cl-0108-2, CLEA Japan, Inc., Tokyo, Japan) with a floor area of 276 mm × 445 mm and 60 g of wood chips (CL-4161, CLEA Japan, Inc.). Mice were acclimatized to the cage for two weeks. During the 2-week acclimation period, two mice were housed in the same cage for the first seven days and individually for the remaining seven days. Mice were fed pellets (CE-2, CLEA Japan, Inc.) and water ad libitum. The feed was set on a wire mesh when the two mice were acclimatizing and placed in a small ceramic dish on the floor during the time of individual acclimation. Flat glass pellets (20 mm diameter) were used as the burrow substrate, and a water bottle (CL-2707, CLEA Japan, Inc.) containing 140 ± 2 g of the burrow substrate was placed on the floor of the cage. Nesting material (5 g; Enviro-dri, Shepherd Specialty Papers, Amherst, MA, USA) was also set on the center of the cage floor. Single housing was used because food intake and nesting and burrowing behaviors were to be evaluated individually. To reduce the stress caused to mice, which are social animals, by single housing, we did not habituate the mice to the experimental environment and single housing at the same time but separated them by one week. Figure 1 shows the outline of the experimental schedule. The Institutional Animal Care and Use Committee of the Okayama University of Science approved this study (approval number: 2021-021).

### 2.2. Treatment Groups

Mice were randomized (using a random number table) into six groups, with seven mice per group. The sample size calculation for repeated-measures analysis of variance (ANOVA) (effect size of 0.7, significance level of 5%, and power of 0.80) estimated seven mice in each group. Groups were designated as: (1) nonsurgical + 0.1 mg/kg buprenorphine + 0 mg/kg meloxicam (N + B + M0); (2) nonsurgical + 0.1 mg/kg buprenorphine + 2.5 mg/kg meloxicam (N + B + M2.5); (3) nonsurgical + 0.1 mg/kg buprenorphine + 5 mg/kg meloxicam (N + B + M5); (4) surgical + 0.1 mg/kg buprenorphine + 0 mg/kg meloxicam (S + B + M0); (5) surgical + 0.1 mg/kg buprenorphine + 2.5 mg/kg meloxicam (S + B + M2.5); and (6) surgical + 0.1 mg/kg buprenorphine + 5 mg/kg meloxicam (S + B + M5). In nonsurgical groups, mice were anesthetized and administered analgesics, but no surgery was performed. In the surgical groups, mice were anesthetized and administered analgesics, and surgery was performed. In the N + B + M0 group and the S + B + M0 group, mice were injected with injectable sterile water instead of meloxicam (Table 1).

### 2.3. Surgery and Perioperative Care

As in our previous study [13], the surgical procedure in this study simulated vas deferens ligation surgery. We assumed that the animals would be used as vas deferens ligation males after three weeks of surgery; five-week-old mice were obtained, and after two weeks of acclimatization, surgical procedures were performed on seven-week-old mice.

Using a 27-gauge needle (Terumo Corporation, Tokyo, Japan), 0.1 mg/kg buprenorphine (Otsuka Pharmaceutical Co., Ltd., Tokyo, Japan), and meloxicam (Kyoritsu Pharmaceutical Co., Ltd., Tokyo, Japan) were subcutaneously (SC) injected to mice 1 h before the start of surgery. Meloxicam dosages were 0, 2.5, or 5 mg/kg. Buprenorphine and meloxicam were administered SC as a mixed solution at 0.1 mL/10 g. Then, using a 26-gauge needle (Terumo Corporation), 0.2 mg/kg medetomidine (Kyoritsu Pharmaceutical Co., Ltd.) at 0.1 mL/10 g was injected intraperitoneally (IP) into mice 15 min before the induction of anesthesia. After checking the sedation induced by medetomidine, mice were placed into an induction chamber (KN-1071 NARCOBIT-E; Natsume Manufacturing Co., Ltd., Tokyo, Japan) and anesthetized with 3–3.5% (flow rate 5000 mL/min) isoflurane in air (Pfizer Inc., Tokyo, Japan) until the righting reflex was lost. Air was used because the anesthesia machine had a built-in air pump. Then, mice were maintained under general anesthesia with 2–2.5% (flow rate 200 mL/min) isoflurane in air using a nose cone until the end of surgery. From the initiation of anesthesia until the mice were returned to the cages in the animal room after surgery, they were kept warm on a warming plate. During anesthesia, the eyes of mice were protected with artificial tears (Senju Pharmaceutical, Osaka, Japan). The surgical site in the lower abdomen was shaved, and the skin of the surgical site was disinfected. After confirming the loss of the pedal withdrawal reflex, an incision (1.5 cm) was made in the midline skin and muscle (rectus abdominis), approximately 1 cm rostral from the penis. The testes, epididymis, and ductus deferens were exposed outside the body for 3 min and returned to the abdominal cavity. After this procedure was performed bilaterally, the muscle was sutured, and a few drops of 0.5% bupivacaine (Aspen Japan Co., Ltd., Tokyo, Japan) were placed in the sutured rectus abdominis area. The skin was then closed with skin staples. Using a 26-gauge needle (Terumo Corporation), 1 mg/kg of atipamezole (Kyoritsu Seiyaku Corporation) at 0.1 mL/10 g was injected IP into mice after surgery. The same experimenter performed all surgeries. After the discontinuation of isoflurane administration, the mice were allowed to recover from anesthesia. After confirming the return of the righting reflex, mice remained on the warming plate for 30 min and were then returned to their cages. Eight hours after the first administration, a second dose of 0.1 mg/kg buprenorphine at 0.1 mL/10 g was administered SC to mice. In addition, meloxicam at 0.1 mL/10 g was administered SC to mice at 9:00 AM on day 2 post-treatment at the same dose as on day 1 post-treatment (Figure 1 and Table 1). The conditions of postoperative mice were checked when they were returned to the cage after 30 min of warming (around 11:30 AM on day 1 post-treatment), when the second dose of buprenorphine was administered (around 5:00 PM on day 1 post-treatment), when the second dose of meloxicam was administered (around 9:00 AM on day 2 post-treatment), and when the burrowing device was placed (5:00 PM on day 2 post-treatment). The checked parameters were the appearance (coat staring, hunching up, and eye and nose discharge), the condition of the stapled closed area (bleeding and whether or not the staples were removed), and the behavior (restlessness, stillness, or self-mutilation in the stapled closed area).

### 2.4. Data Collection

Using the methods described in our previous report [13], food intake and body weight were measured, and nest consolidating and burrowing behaviors were assessed. Frequent handling of the mice could cause stress. Therefore, the measurement of body weight and food intake, the placement of the water bottle containing glass pellets and nest materials, and the administration of analgesics were conducted at the same time of day in the morning and evening. In addition, to reduce frequent contact with the cage, burrowing and nest-building behaviors were recorded using a network camera and then analyzed later.

#### 2.4.1. Food Intake

The three pellets placed in a small pottery plate were changed daily at 9:00 AM. Pellet weights were measured from the pretreatment day until day 3 post-treatment (Figure 1). Differences between the weight of provided pellets and that of the remaining pellets at the time of change were used to estimate the food intake. The value at 9:00 AM on day 1 post-treatment (before administration of buprenorphine and meloxicam) subtracted from the value at 9:00 AM on the pretreatment day was used as the baseline value for food intake. The value at 9:00 AM on day 2 post-treatment (before administration of meloxicam) subtracted from the value at 9:00 AM on day 1 post-treatment was used as the food intake value 24 h after treatment, and the value at 9:00 AM on day 3 post-treatment subtracted from the value at 9:00 AM on day 2 post-treatment was used as the food intake value 48 h after treatment. The percent change in food intake at 24 and 48 h after treatment was calculated by dividing the measured values by the baseline values. The percentages of the baseline values were calculated by dividing each of the measured values by the mean.

#### 2.4.2. Body Weight

The body weights of mice were taken daily at 9:00 AM from day 1 post-treatment to day 3 post-treatment at 24 h intervals. The body weight on day 1 post-treatment (before administration of buprenorphine and meloxicam) was the baseline value. The value on day 2 post-treatment (before administration of meloxicam) was used as the body weight at 24 h after treatment, and the value on day 3 post-treatment was used as the body weight 48 h after treatment. The percent changes in body weight at 24 and 48 h after treatment were calculated by dividing the measured values by the baseline values. The percentages of the baseline values were calculated by dividing each of the measured values by the mean.

#### 2.4.3. Nest Consolidating Behavior

**Figure 2:**
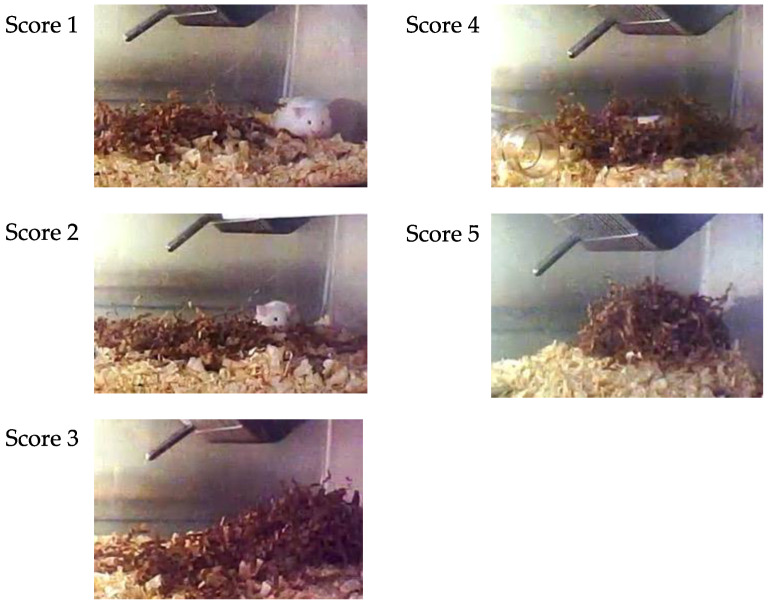
Representative images of nest consolidation scores.

The nest consolidating behavior of the mice was recorded using a network camera (Qwatch TS-WRLP, I-O Data Device, Inc., Ishikawa, Japan) from the pretreatment day to day 3 post-treatment. Nest materials were disentangled and placed back in the center of the cage floor at 9:00 AM daily, and the nest consolidation score was assessed at 2, 4, 6, 8, 12, 16, and 24 h after the nest materials were disentangled and positioned. The same nesting material was used repeatedly. Only on day 1 post-treatment, the time when treated mice were returned to their cages was set as the time of nest material placement. The nest consolidation score was assessed on a 5-point scale: 1 = no nesting sites formed, the mouse does not use nesting material; 2 = no nesting sites formed, the mouse rides on or crawls under nesting material; 3 = no nesting sites formed, the mouse collects some nesting material, but the shape of nesting material is incomplete and flat; 4 = nest sites formed, the nest is cup-shaped; 5 = nest sites formed, the nest form is an incomplete dome- or dome-shaped (Figure 2). The nest consolidation score was modified with reference to Hess et al. [23].

#### 2.4.4. Burrowing Behavior

Mice burrowing behavior was recorded using a network camera from the pretreatment day to day 3 post-treatment. A water bottle containing glass pellets was placed in the cage as a burrowing device. Reset was defined as placing all glass pellets into the water bottle, and resetting was performed at 5:00 PM. The latency to burrow was defined as the removal of more than three glass pellets from the device within 10 s [18,24]. The latency to burrow was measured in seconds.

### 2.5. Histological Assessment of the Stomach, Duodenum, and Jejunum

Under deep anesthesia with 3.5–4% isoflurane, mice were abdominally incised, and the abdominal aorta and posterior vena cava were cut. After confirming cardiac arrest, the stomach, duodenum, and jejunum were sampled. The duodenum and jejunum were rolled longitudinally to form Swiss roll samples. Tissues were fixed in 10% buffered formalin, paraffin-embedded, sectioned, and stained with hematoxylin and eosin. Similar sections of the stomach, duodenum, and jejunum of all mice were evaluated histologically.

### 2.6. Statistical Analysis

All statistical analyses were performed using the Prism 9 software program (GraphPad Software, San Diego, CA, USA). The change in food intake and body weight between groups were compared using repeated-measures two-way ANOVAs, and Tukey’s post hoc test was conducted. The nest consolidation scores were compared between groups using the Kruskal–Wallis test, and Dunn’s post hoc test was conducted. The distribution of latency to burrow was examined using Kaplan–Meier survival analysis. The log-rank test was conducted to assess whether latency to burrow differed statistically between groups. In all the tests, the significance was set at *p* < 0.05.

## 3. Results

### 3.1. Changes in Food Intake

**Figure 3:**
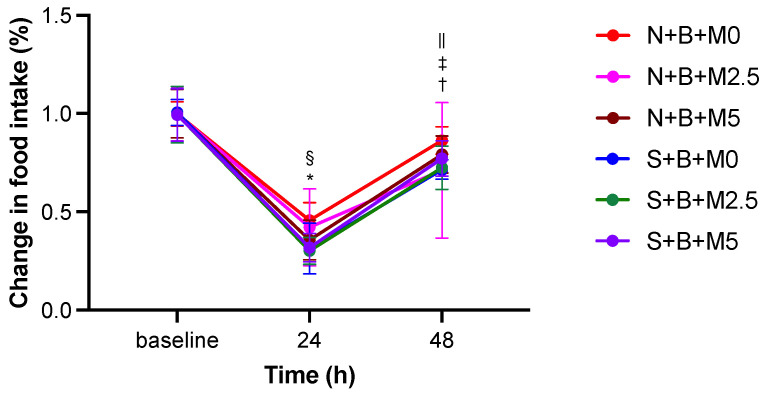
Comparison of post-treatment changes in food intake. Data are presented as mean ± SD. *, value significantly differs (*p* < 0.01) between the baseline and 24 h in all groups. †, value significantly differs (*p* < 0.01) between 24 h and 48 h in all groups. ‡, value significantly differs (*p* < 0.05) between the baseline and 48 h (excluding the N + B + M0, N + B + M2.5, and N + B + M5 groups). §, value significantly differs (*p* < 0.05) between the N + B + M0 and S + B + M2.5 groups. ||, value significantly differs (*p* < 0.03) between the N + B + M0 and S + B + M0 groups. N + B + M0 group: *n* = 6; N + B + M2.5 group: *n* = 6; N + B + M5 group: *n* = 6; S + B + M0 group: *n* = 7; S + B + M2.5 group: *n* = 7; and S + B + M5 group: *n* = 7.

Food intake data 48 h after treatment for the N + B + M0, N + B + M2.5, and N + B + M5 groups were analyzed at *n* = 6 because of missing data for one animal, whereas data for the other groups were analyzed at *n* = 7. In repeated-measures two-way ANOVA, the interaction between group and time was not significant (F (10,70) = 0.75), but there was a main effect of time (*p* < 0.01). In all groups, food intake was significantly decreased (*p* < 0.01) at 24 h after treatment relative to the baseline, and food intake significantly increased (*p* < 0.01) at 48 h after treatment compared with that at 24 h after treatment. However, except for the N + B + M0, N + B + M2.5, and N + B + M5 groups, food intake at 48 h after treatment did not recover to the baseline level (Figure 3). At 24 h after treatment, food intake in the N + B + M0 group was significantly higher than that in the S + B + M2.5 group. At 48 h after treatment, food intake in the N + B + M0 group was significantly higher than that in the S + B + M0 group.

### 3.2. Changes in Body Weight

**Figure 4:**
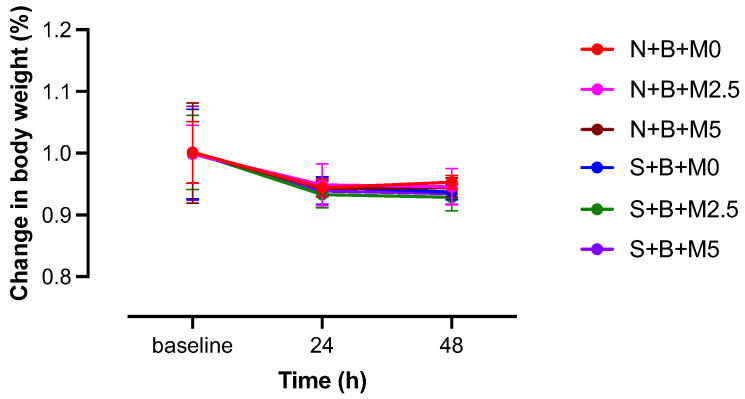
Comparison of post-treatment changes in body weight. Data are presented as mean ± SD. N + B + M0 group: *n* = 7; N + B + M2.5 group: *n* = 7; N + B + M5 group: *n* = 7; S + B + M0 group: *n* = 7; S + B + M2.5 group: *n* = 7; and S + B + M5 group: *n* = 7.

In repeated-measures two-way ANOVA, the interaction between group and time was not significant (F (10,72) = 0.1021). Although not statistically significant, the body weight in all groups showed a decreasing trend at 24 h after treatment compared with that at the baseline, and body weight at 48 h after treatment shifted to the same level as that at 24 h after treatment (Figure 4).

### 3.3. Nest Consolidating Behavior

Kruskal–Wallis test results showed that the N + B + M0 group scored significantly higher than the N + B + M2.5 group 2 h after setting the nest material on the pretreatment day (*p* < 0.03) and that the S + B + M5 group scored significantly higher than the N + B + M0 group 2 h after setting the nest material on day 2 post-treatment (*p* < 0.03). Otherwise, no differences in nest consolidation scores were observed among any groups (Table 2).

### 3.4. Burrowing Behavior

**Figure 5:**
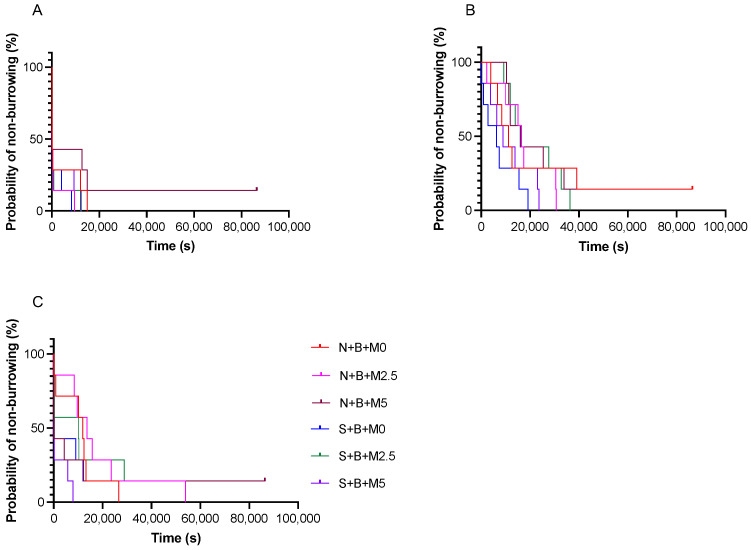
Kaplan–Meier analysis of the latency to burrow: (**A**) Day pretreatment. (**B**) Day 1 post-treatment. (**C**) Day 2 post-treatment. N + B + M0 group: *n* = 7; N + B + M2.5 group: *n* = 7; N + B + M5 group: *n* = 7; S + B + M0 group: *n* = 7; S + B + M2.5 group: *n* = 7; and S + B + M5 group: *n* = 7.

There were no significant differences in the latency to burrow among any groups on the day before treatment and on day 1 post-treatment (Figure 5A,B). On day 2 post-treatment, mice showed significantly longer latencies to burrow in the N + B + M0, N + B + M2.5, and S + B + M2.5 groups (*p <* 0.05; Figure 5C).

### 3.5. Histological Assessment of the Stomach, Duodenum, and Jejunum

On histopathological evaluation, no morphological abnormalities were found in the stomach, duodenum, and jejunum of any animals (data not shown).

## 4. Discussion

In a study using the grimace scale to evaluate the analgesic effect on mice subjected to laparotomy [25], a relatively high level of pain was observed for 8–12 h postoperatively, and the pain was suggested to be present for 36–48 h postoperatively. Therefore, we administered analgesics to mice for 48 h postoperatively and evaluated the effects of a multimodal analgesic protocol using buprenorphine with meloxicam on the well-being of mice during analgesic administration by changing the dosage of meloxicam, under the hypothesis that a multimodal analgesic protocol using buprenorphine with meloxicam has analgesic effects.

Buprenorphine administration has been reported to reduce food intake and body weight in postoperative mice [7,8,16,26,27]. It has also been reported that meloxicam administration reduces body weight in postoperative mice [28] and that isoflurane administration decreases food intake in mice [29]. In addition, the multimodal approach has been reported to reduce food intake and body weight [7,8,13]. In all study groups, there was a significant decrease in food intake 24 h after treatment but a significant increase 48 h post-treatment. Body weight in all groups showed a decreasing trend but not a significant reduction. Furthermore, the histological evaluation of the stomach, duodenum, and jejunum showed no morphological abnormalities. We found no previous reports indicating that the subcutaneous administration of 5 mg/kg meloxicam causes morphological abnormalities in the gastrointestinal tract of mice in a few days; moreover, no stomach or duodenal NSAID-related pathologies were observed in a study in which 20 mg/kg meloxicam was subcutaneously administered to mice for 6 days [30]. Thus, it is unlikely that the decrease in food intake is due to meloxicam-induced organic abnormalities in the gastrointestinal tract. These results suggest that when buprenorphine is combined with meloxicam (up to 5 mg/kg/day for 48 h) postoperatively, only a minimal reduction in food intake and body weight is to be expected in mice.

There were significant differences in nest consolidation scores between some groups (N + B + M0 vs. N + B + M2.5 and N + B + M0 vs. S + B + M5) on the pretreatment day at 2 h and on day 2 post-treatment at 2 h. Otherwise, there were no significant differences. Moreover, significant differences were observed in the latency to burrow between the N + B + M0, N + B + M2.5, and S + B + M2.5 groups on day 2 post-treatment. Otherwise, there were no significant differences. These significant differences among some of the groups found in nest consolidation scores and the latency to burrow were not considered indicative of an effect of the surgery and/or the meloxicam dose.

Because there was no surgical group without analgesics in this study, we cannot completely rule out the possibility that postoperative pain did not cause a reduction in food intake and delay in the onset of nesting behavior. However, it has been widely reported that surgery-inflicted pain causes weight loss [8], decreased food intake [17,31], and prolonged nest consolidation and latency to burrow [8,17,18,20,21,32]. In our study, there were no significant differences in food intake, nest consolidation score, or latency to burrow among any groups, and no effects associated with the surgical procedure were observed. Thus, it is possible that all analgesic combinations used had an analgesic effect.

When a tissue is traumatized, various pain-related chemical mediators are released, enhancing inflammatory responses and activating nociceptors [1]. The continued input of pain information to the central nervous system because of inadequate pain management can cause hyperalgesia and allodynia [1]. Meloxicam, a member of the oxicam family of NSAIDs, inhibits the synthesis of prostaglandins, which are pain-related chemical mediators [1]. The results of our study suggest that the negative effects of meloxicam administration on the well-being of mice are likely to be minimal. Because meloxicam administration decreases sensitivity to pain, it can be used in combination with buprenorphine, an opioid that inhibits neuronal excitability, to provide more options for the well-being of mice after abdominal surgery. While some reports recommend using multimodal approaches for rodents [8,9,33], adverse effects have also been reported [7,8]. The surgical implantation of a radiotelemetry device into the abdominal cavity is associated with abdominal pain that can easily affect food intake and is more likely to impact body weight and growth rate than other surgical procedures [7,34]. The use of a multimodal approach, in this case, requires special attention because of its greater impact on weight loss and reduced food intake [7,8,13]. Although preemptive analgesia and multimodal analgesia contribute to minimizing postoperative animal stress and optimizing well-being [4], multimodal approaches are not commonly used in rodents, and reports on their effects and influences are lacking.

During postoperative pain management, the administration of analgesics alone is not sufficient to keep the animals comfortable. The influence of the housing environment, social housing, and care (e.g., warmth, soft and dry bedding materials, accessibility of food and water, and palatable food) on postoperative animal recovery should also be considered [6,24,35,36,37,38]. Furthermore, what the laboratory animals experience during handling and treatment may have both positive and negative effects on the experiment. “Handling” includes not only the experimental procedures, such as drug administration and surgery, but also handling in the context of routine maintenance. It has been reported that changes in routine handling can lead to voluntary interaction with humans, which leads to reduced anxiety in mice [39,40,41,42,43]. Such nonpharmacological interventions may help improve the well-being of postoperative animals and should be further investigated along with pharmacological interventions.

There are several limitations to this study. Only a single strain of male mice was used in the experiment. The age of the mice was also limited to 5–7 weeks. We did not investigate the differences in the responses of different mouse strains, genders, or ages, which may lead to different results [22,44]. Because the procedure applied was a median abdominal incision, we did not investigate the effect with other surgical procedures. In this study, mice were individually kept for data collection. The handling method used was picking up mice by the tail. Since housing and handling conditions were similar among all groups, it is unlikely that these factors contributed to the differences in results between groups, but the possibility that these factors stressed the mice and affected their behavior cannot be ruled out. Furthermore, bupivacaine was not administered to the nonsurgical group; thus, its effect could not be compared with that in the surgical group. Since bupivacaine has a duration of up to 8 h [33], its effect was estimated to last from the end of surgery to the evening of day 1 post-treatment, which is the recovery time from anesthesia. We have found no previous reports indicating that bupivacaine possesses an effect on food intake, body weight, or activity levels in mice. On the other hand, bupivacaine absorption is affected by various factors and may not result in analgesia. Thus, bupivacaine administration likely only had a small effect on the food intake, body weight, or behavior of the mice. In this study, the method of meloxicam administration was changed from oral to subcutaneous so that meloxicam and buprenorphine could be administered simultaneously at certain time points, which allowed for a less frequent administration schedule of meloxicam, ultimately contributing to the implementation of the 3Rs principle (replacement, reduction, and refinement) regarding animal use. However, we were not able to evaluate the effect of changing the method of administration of meloxicam (from oral to subcutaneous administration) on the well-being of mice. In addition, the evaluation of the well-being of mice was limited to measurements and observations up to 48 h postoperatively. Moreover, the effect of analgesic monotherapy on pain sensitivity was also not confirmed. Although it is unlikely that the recovery status of animals will deteriorate after 48 h of surgery, to demonstrate the efficacy of a multimodal approach using buprenorphine and meloxicam and the effect of the change in the administration method, the continued evaluation of the well-being of mice after the completion of analgesic administration is necessary.

## 5. Conclusions

When buprenorphine was combined with meloxicam, administering up to 5 mg/kg/day of meloxicam for 48 h to male mice (7 mice per group) after abdominal surgery did not significantly affect food intake, body weight, nest consolidating and burrowing behaviors, or gastric and duodenal tissue histology in a negative manner.

## Figures and Tables

**Table 1:** Drugs administered to each group of mice. Abbreviations in the table; N: nonsurgical group; S: surgical group. B: 0.1 mg/kg buprenorphine administration. M0: 0 mg/kg meloxicam administration; M2.5: 2.5 mg/kg meloxicam administration; M5: 5 mg/kg meloxicam administration. SC: injected subcutaneously. The “drop” in the bupivacaine column indicates that a few drops of 0.5% bupivacaine were placed in the sutured rectus abdominis.

	Treatment	N + B + M0 S + B + M0	N + B + M2.5 S + B + M2.5	N + B + M5 S + B + M5
Day 1 post-treatment	Buprenorphine	0.1 mg/kg (SC) 2 times	0.1 mg/kg (SC) 2 times	0.1 mg/kg (SC) 2 times
	Meloxicam	Sterile water for injection (SC)	2.5 mg/kg (SC)	5 mg/kg (SC)
	Bupivacaine	N + B + M0: None S + B + M0: 0.5% (drop)	N + B + M2.5: None S + B + M2.5: 0.5% (drop)	N + B + M5: None S + B + M5: 0.5% (drop)
Day 2 post-treatment	Meloxicam	Sterile water for injection (SC)	2.5 mg/kg (SC)	5 mg/kg (SC)

**Table 2:** Comparison of nest consolidation scores before and after treatment. Data are presented as median (25–75% percentile). *, value significantly differs (*p* < 0.03) between the N + B + M0 and N + B + M2.5 groups. †, value significantly differs (*p* < 0.03) between the N + B + M0 and S + B + M5 groups. N + B + M0 group: *n* = 7; N + B + M2.5 group: *n* = 7; N + B + M5 group: *n* = 7; S + B + M0 group: *n* = 7; S + B + M2.5 group: *n* = 7; and S + B + M5 group: *n* = 7.

Treatment		N + B + M0	N + B + M2.5	N + B + M5	S + B + M0	S + B + M2.5	S + B + M5
Day pre-treament	2 h	5 (4–5) *	3 (2–5)	3 (2–5)	4 (4–5)	4 (2–5)	4 (3–5)
	4 h	5 (5–5)	4 (3–5)	4 (3–5)	5 (4–5)	5 (3–5)	4 (3–5)
	6 h	5 (5–5)	4 (4–5)	4 (3–5)	5 (5–5)	5 (3–5)	4 (4–5)
	8 h	5 (5–5)	4 (4–5)	4 (4–5)	5 (5–5)	5 (3–5)	5 (4–5)
	12 h	5 (5–5)	4 (4–5)	4 (4–5)	5 (5–5)	5 (3–5)	5 (4–5)
	16 h	5 (5–5)	5 (5–5)	4 (4–5)	5 (5–5)	5 (3–5)	5 (5–5)
	24 h	5 (5–5)	5 (5–5)	5 (5–5)	5 (5–5)	5 (3–5)	5 (5–5)
Day 1 post-treatment	2 h	1 (1–1)	1 (1–1)	1 (1–2)	1 (1–2)	1 (1–2)	1 (1–2)
	4 h	1 (1–2)	1 (1–1)	1 (1–2)	1 (1–2)	2 (1–2)	1 (1–2)
	6 h	2 (1–3)	1 (1–3)	1 (1–2)	2 (1–3)	2 (1–2)	2 (1–3)
	8 h	2 (2–3)	1 (1–3)	1 (1–2)	2 (1–3)	2 (1–2)	2 (2–3)
	12 h	3 (2–3)	2 (1–3)	2 (1–3)	2 (2–4)	2 (2–3)	3 (2–4)
	16 h	3 (3–5)	4 (3–5)	3 (2–5)	3 (2–5)	3 (3–4)	5 (4–5)
	24 h	5 (3–5)	5 (4–5)	4 (2–5)	5 (2–5)	4 (3–5)	5 (4–5)
Day 2 post-treatment	2 h	2 (2–2)	3 (2–4)	3 (2–4)	2 (2–3)	3 (2–4)	4 (3–5) †
	4 h	2 (2–4)	3 (2–4)	3 (2–5)	3 (2–4)	4 (2–5)	4 (4–5)
	6 h	4 (3–4)	3 (2–4)	3 (2–5)	3 (2–4)	4 (2–5)	5 (4–5)
	8 h	4 (3–4)	3 (3–4)	3 (3–5)	3 (2–4)	4 (3–5)	5 (4–5)
	12 h	4 (3–4)	3 (3–4)	4 (3–5)	3 (2–5)	4 (3–5)	5 (4–5)
	16 h	4 (4–5)	4 (3–4)	5 (4–5)	4 (3–5)	4 (3–5)	5 (4–5)
	24 h	5 (4–5)	4 (4–5)	5 (5–5)	5 (3–5)	5 (5–5)	5 (4–5)

## Data Availability

Not applicable.
